# Incorporating the Detection of Single Nucleotide Polymorphisms Associated With Invasive Aspergillosis Into the Clinic

**DOI:** 10.3389/fcimb.2022.860779

**Published:** 2022-04-12

**Authors:** P. Lewis White, Jessica S. Price

**Affiliations:** Public Health Wales Mycology Reference Laboratory, University Hospital of Wales (UHW), Cardiff, United Kingdom

**Keywords:** invasive aspergillosis, predictive models, clinical risk, genetic risk, SNPs

## Abstract

Exposure to fungi is inevitable, yet only a small number of patients with significant clinical risk develop invasive aspergillosis (IA). While timing of exposure in relation to immune status, environmental and occupational factors will influence the probability of developing IA, factors specific to the individual will likely play a role and variation in the host’s genetic code associated with the immunological response to fungi have been linked to increased risk of developing IA. Screening for SNPs in genes significantly associated with IA (e.g. Pentraxin-3, Toll-like receptor 4, Dectin-1, DC-SIGN) could form part of the clinical work-up on admission or post allogeneic stem cell transplantation, to complement fungal biomarker screening. Through the combination of clinical and genetic risk with mycological evidence, we are approaching a time when we should be able to accurately predict the risk of IA in the haematology patient, using predictive modelling to stratifying each individual’s management. Understanding the host and their immune responses to infection through genomics, transcriptomics and metabolomics/proteomics is critical to achieving how we manage the individual’s risk of IA, underpinning personalized medicine. This review will investigate what is known about the genetic risk associated with developing IA, primarily in haematology patients and whether these strategies are ready to be incorporated into routine clinical practice, and if not what are the remaining hurdles to implementation.

## Introduction

The exposure of the human being to fungi, whether environmental or commensal, is inevitable. Yet most fungi only demonstrate a pathogenic capacity when permitted conditions within the host that are preferential to fungal proliferation (e.g. immunosuppression or disruption to anatomical barriers). The number of patients requiring immunosuppressive conditioning increases annually, and despite many patients having similar degrees of clinical risk for developing invasive fungal disease (IFD), only a relatively small proportion develop IFD despite the inevitable exposure to fungi, which itself is very difficult to control or obviate.

Why certain patients develop IFD remains unclear. In simple terms, luck may play a part, where the timing of exposure to a significant fungal burden does not coincide with a period of increased clinical risk (e.g. prolonged, profound neutropenia) of developing IFD. In the unlucky few, enhanced clinical risk corresponds with exposure to high fungal burdens due to local environmental conditions, close proximity to construction or even occupational hazards. The virulence of the infecting organism and subsequent exposure to such a strain may be significant. Certain strains of *Aspergilllus* may have an increased capacity for causing infection, however, a recent UK and Ireland epidemiological study using next generation sequencing determined that clinical strains were intrinsically linked to those circulating in the environment [Rhodes et al., (Forthcoming)].

Obviously clinical management can minimize the risk of developing IFD, with antifungal prophylactic strategies widely employed in high risk groups (e.g. acute leukaemia or allogeneic stem cell recipients) and can successfully lower the incidence of IFD [e.g. invasive aspergillosis (IA)] (Wang et al., 2020). Nevertheless, even in the absence of antifungal prophylaxis the majority (>85%) of high risk haematology patients will not develop IFD/IA, despite the apparent risk and inevitable fungal exposure (Barnes et al., 2018).

This indicates other factors may play a role in governing the risk of developing IFD in the individual patient already considered clinically at high-risk of IFD. Genetic variants, specifically single nucleotide polymorphisms (SNPs), in the host’s genetic code associated with the innate immunological response to fungi have been linked to increased risk of developing IA (Cunha and Carvalho, 2019). Given an individual’s genetic code is unique, increased risk of IFD due to the presence of genetic polymorphisms provides a reason as to why only certain patients develop IFD. Prior knowledge to the presence of these polymorphisms within the specific patient could be used to stratify risk of IFD on an individual basis (personalized medicine). Screening for SNPs could form part of the clinical work-up on admission or post allogeneic stem cell transplantation, to complement fungal biomarker screening.

Despite our increasing knowledge on the importance of SNPs in regards to developing IFD, in particular IA, approaches to implement such tests in prospective practice remain limited (Tanpaibule et al., 2020). This review will investigate what is known about the genetic risk associated with developing IA and whether these strategies are ready to be incorporated into routine clinical practice, and if not what are the remaining hurdles to implementation.

## Understanding Test Utility

The primary functions of any testing are to either exclude or confirm a diagnosis. While in principle a single test has the potential to facilitate both parameters, in practice, the pre-requisite high sensitivity required to exclude infection when the test is negative usually compromises specificity to below levels necessary for confidence in a positive result required to confirm infection. With the incidence of IA generally being low (<10%), the pre-test probability of not having IA is high (>90%) and can be exploited by mycological testing [combined *Aspergillus* PCR and Galactomannan EIA (GM)] to exclude infection when both tests are consistently negative (Arvanitis et al., 2015). The combined screening strategies are employed to provide sufficient sensitivity and also have the capacity to increase confidence in diagnosis through the availability of sequential/multiple positive biomarker results, but even when a patient is positive on multiple occasions by both PCR and GM, the post-test probability is not always convincing of infection (55%) (Barnes et al., 2013). Confidence in infection can be enhanced by performing a diagnostic work-up (Bronchoscopy/HRCT Chest) of any high-risk patient with multiple positive biomarker results, but these additional investigations are not always performed in a timely manner, and waiting on these results to commence treatment is not recommended given the deleterious impact of delaying necessary antifungal therapy.

As with all clinical conditions “prevention is better than a cure”, to achieve this, action is taken against an anticipated outcome, based on the odds of developing future disease. The odds are influenced by various variables, including clinical conditions, environmental exposure and potentially genetic predisposition to infection. Combining these risk factors into a strategic algorithm, also incorporating existing biomarker screening has the potential to provide a personalized pre-emptive approach, where various thresholds can be set to change patient management (e.g. continue to monitor, antifungal prophylaxis, pre-emptive therapy, targeted therapy) based on the probability of developing IA.

Empirical antifungal therapy (treating a fever refractory to broad spectrum antibiotics) is widely administered in high-risk hematology patients. This approach was incorporated in an era when access to enhanced mycological testing was not available and this strategy bases the risk of developing IA solely on underlying clinical risk, leading to many patients receiving unnecessary antifungal therapy which is far from ideal given the increasing rates of antifungal resistance and the adverse effects/drug-drug interactions associated with antifungal drugs. Biomarkers can be early indicators of infection in hematology patients with IA. Positivity permits the opportunity to pre-empt overt disease, provided biomarker screening does not generate excessive numbers of false positive results, but the current post-test probability of IA associated with multiple positives remains insufficient for purpose (Barnes et al., 2013; Aguado et al., 2015; Cruciani et al., 2019). Combining biomarkers with clinical risk and data on genetic predisposition to IA has the potential to significantly improve how we manage patients at risk of IA.

## Genetic variants associated with invasive aspergillosis and barriers to routine clinical use

An extensive number of SNPs have been linked to increased susceptibility of developing IA, where genetic variations in the genes encoding pattern recognition receptors [PRRs, e.g. toll like receptors (TLR) or C-type lectin receptors] alter the signalling pathway of the host’s innate immune response, but a wide array of genetic variations in other genes encoding innate immunity components have been documented, along with genetic variation in components of the host’s adaptive immunity (e.g. Th-17 or IFN-γ, **Table 1**) (van der Velden et al., 2011; Sainz et al., 2012; Lupiañez et al., 2015; Fisher et al., 2017; Cunha et al., 2018; Cunha and Carvalho, 2019).

**Table 1:** A summary of selected genetic variants associated with aspergillosis in various patient cohorts.

Protein type		Haplotype/polymorphism or Amino acid substitution	Functional impact	Clinical validation (REF)
TLR	TLR1	N248S	Potentially effecting the production of circulating cytokines	Increased susceptibility to IA in SCT patients with polymorphism (Kesh et al., 2005).
	TLR2	R753Q	Impairs signalling competent and downstream innate immunological response	Increased susceptibility to pneumonia including IFD in AML patients (Fischer et al., 2016).
	TLR3	C>A nucleotide substitution at position 95	Decreased expression and responsiveness of receptor compromising CD8 T-cell responses	Increased susceptibility to IA in SCT patients with polymorphism (Carvalho et al., 2012).
	TLR4	D299G, T399I	Reduced Interaction between receptor lipopolysaccharide target	Increased incidence of IA in allogeneic SCT pts with polymorphism (Bochud et al., 2008). Increased incidence of chronic aspergillosis (Carvalho et al., 2008).
	TLR5	R392X	Early stop codon	Increased susceptibility to IA in SCT pts with polymorphism (Grube et al., 2013).
	TLR6	S249P	Potentially effecting the production of circulating cytokines	Increased susceptibility to IA in SCT patients with polymorphism (Kesh et al., 2005).
CLR	Dectin-1	Y238X	Truncated receptor with reduced ability to bind BDG	Increased susceptibility to IA in SCT patients with polymorphism (Cunha et al., 2010).
		C>G nucleotide substitution at rs7309123	NA	Increased susceptibility to IA in hematology patients with polymorphism (Sainz et al., 2012).
		G>T nucleotide substitution at rs3901533	NA	Increased susceptibility to IA in hematology patients with polymorphism (Sainz et al., 2012).
	Dectin-2	Base pair deletion (507del C) resulting in early stop codon	Functional defective - Reduced levels of protein, which do not cluster nor bind/respond *A. fumigatus*	Case report of IA (Griffiths et al., 2021)
	DC-SIGN	A>G nucleotide substitution at rs4804800	Affects RNA expression	Increased susceptibility to IA in hematology patients with polymorphism (Sainz et al., 2012).
		C>T nucleotide substitution at rs11465384	Affects RNA expression	Increased susceptibility to IA in hematology patients with polymorphism (Sainz et al., 2012).
		G>C nucleotide substitution at rs7252229	NA	Increased susceptibility to IA in hematology patients with polymorphism (Sainz et al., 2012).
		(A>G nucleotide substitution at rs7248637)	Affects RNA expression	Increased susceptibility to IA in hematology patients with polymorphism (Sainz et al., 2012).
	CARD-9	Q295X and Absent protein (G>C polymorphism in the start codon)	Reduced production of neutrophil chemo-attractants	Case reports of extra-pulmonary aspergillosis (Rieber et al., 2016).
	MelLec	G26A	Impaired cytokine production, likely due to reduced intracellular signal transduction.	Increased susceptibility to IA in SCT patients with polymorphism (Stappers et al., 2018).
Others, including soluble PRR, cytokines/chemokines, proangiogenic factors	MBL	NA	Lowered circulated MBL concentrations.	Low NBL concentrations determined in patients with IA (Lambourne et al., 2009)
	PTX3	rs2305619 A>G (GG genotype) rs3816527 A>C (AA genotype) H2/H2 (GA/GA Haplotype)	Defective alveolar expression of PTX3 leads to deficiency in neutrophils, impaired phagocytosis and fungal clearance	Increased susceptibility to aspergillosis in SCT, SOT and COPD patients with polymorphism (Cunha et al., 2014; Cunha et al., 2015; Wójtowicz et al., 2015; Fisher et al., 2017; He et al., 2018).
	PLG	D472N	Altered structure of ligand binding/kringle domains – potentially enhancing binding of *A. fumigatus*, increasing PLG activation and facilitating pathogen tissue entry and damage	Increased susceptibility to IA in SCT patients with polymorphism (Zaas et al., 2008).
	RAGE	-374 T>A	Enhanced expression of pro-inflammatory cytokines (IL-17A and IFN-γ) contributing to progression of IA	Increased susceptibility to IA in SCT patients with polymorphism (Cunha et al., 2011).
	CXCL10	rs1554013 C>T rs3921 C>G rs4257674 A>G	Reduced CXCL10 expression which impacts the subsequent T-cell response to *Aspergillus.*	Increased susceptibility to IA in SCT patients with polymorphism (Mezger et al., 2008).
	TNFR1	rs4149570 G>T rs767455 A>G	Reduced mRNA levels expression, potentially modifying NFkappaB signalling pathway	Increased susceptibility to IA in hematology patients with polymorphism (Sainz et al., 2010).
	IFN-γ+TLR4	IFNG 874t>A TLR4 1063A>G	IFNG 874t>A: Sub-optimal production of IFNG, decreased macrophage activation. TLR4 1063A>G Reduced Interaction between receptor lipopolysaccharide target	Increased susceptibility to IA in SCT patients with haplotype (de Boer et al., 2011).
	S100B	427 C>T	Increased S100B secretion in serum, when in excess progressing infection *via* the RAGE pathway	Increased susceptibility to IA in SCT patients with polymorphism (Cunha et al., 2011).
	ARNT2 CX3CR1	ARNT2 rs1374213G CX3CR1 rs7631529A CX3CR1 rs9823718G CX3CR1 AGCGG Haplotype	Impaired fungicidal activity and deregulated immune responses of monocyte derived macrophages	Increased susceptibility to IA in hematology patients with polymorphisms (de Boer et al., 2011).
	IL-10	ATA haplotype rs1800896, GG genotype	Polymorphism in the promoter region leads to higher amounts of IL-10 Polymorphism leads to higher expression of IL-10 mRNA and protein expression leading to 25% reduction in fungal clearance. PBMCs with the GG genotype also secreted lower amounts of TNF-α potential lessening inflammatory responses	Increased susceptibility to IA in SCT patients with polymorphism (Seo et al., 2005). Increased susceptibility to IA in SCT patients with polymorphism from donor in a two phase study (Cunha et al., 2017).
	TNFSF4 MAPKAPK2	rs7256628T/T rs12137965G	Decreased levels of TNFSF14 protein and macrophages with decreased antifungal activity. Increased numbers of CD38+IgM IgD- plasmablasts in blood or decreased serum concentrations of thymic stromal lymphopoietin	Increased risk of developing IA in hematology patients with the polymorphisms (Sánchez-Maldonado et al., 2020).
	STAT3	STAT-3 Mutations (R382W, S560del, V637M, I568F, S668Y)	Lower concentrations of adaptive cytokines (IFN-γ, IL-17, IL-22) associated with STAT3 deficiency	Aspergillosis (chronic/allergic) associated with a defective adaptive immune response (Danion et al., 2020).
	NOD2	rs2066842, P268S	Absence of NOD2 in monocytes and macrophages increases phagocytosis and fungal killing	Decreased risk of IA after SCT (Gresnigt et al., 2018)
	IL4R IL8 VEGFA IFN-γ IL-12B	rs2107356A/A rs2227307G/G rs2146323A rs6900017T rs2069705C rs3212227C	NA NA NA NA Macrophages with Increased ability to kill fungal spores, increased mRNA expression elevating IFN-γ, IL12p70 and TNF levels	Increased risk of developing IA in hematology patients with the polymorphisms (Lupiañez et al., 2015). Decreased risk of developing IA in hematology patients with the polymorphisms (Lupiañez et al., 2015).

TLR, Toll like receptor; IA, Invasive aspergillosis; SCT, Stem cell transplantation; IFD, Invasive fungal disease; AML, Acute myeloid leukemia; CD8, Cluster of differentiation 8; CLR, C-type lectin receptors; BDG [Rhodes et al., (Forthcoming); Barnes et al., 2018; Wang et al., 2020],-β-D-glucan; NA, Not available; DC-SIGN, Dendritic Cell-Specific Intercellular adhesion molecule; RNA, Ribonucleic acid; CARD9, Caspase recruitment domain-containing protein 9; MelLec, Melanin sensing C-type Lectin receptor; MBL, Mannan-binding lectin; PRR, Pattern recognition receptors; PTX3, Pentraxin 3; SOT, Solid organ transplantation; COPD, Chronic obstructive pulmonary disease; PLG, Plasminogen; RAGE, Receptor for advanced glycation end products; IL, Interleukin; IFN-γ, Interferon-gamma; CXCL10, C-X-C motif chemokine ligand 10; TNFR, Tumor necrosis factor receptor;mRNA, messenger Ribonucleic acid; NFkappaB, Nuclear factor kappa B; S100B, S100 calcium binding protein; ARNT2, Aryl Hydrocarbon Receptor Nuclear Translocator 2; CX3CR1, CX3C chemokine receptor 1; PBMC, Peripheral blood mononuclear cells; TNF-α, Tumor necrosis factor – alpha; TNFSF4, Tumor necrosis factor super family member 4; MAPKAPK2, MAPK Activated Protein Kinase 2; STAT3, Signal transducer and activator of transcription 3; NOD, Nucleotide-binding oligomerization domain; VEGFA, V*ascular *Endothelial Growth Factor A.**

The significant number of polymorphisms linked to increased susceptibility reflects the complexity of the host’s immune response to *Aspergillus* and screening for such a large number of polymorphisms is a major challenge when attempting to incorporate genetic testing into routine strategic algorithms. While next generation sequencing surely holds the key for screening hosts or donors for polymorphism associated with aspergillosis (and will undoubtedly highlight additional genetic markers), further work is needed to elucidate the associated phenotypic alterations to protein structure, function and implications to any immunological pathways involving such proteins. Not all genetic variants have been functionally characterized, and it is essential this is addressed to develop an enhanced understanding of the host’s defense to IA. Further knowledge regarding the presence of multiple polymorphism’s within the same, but more so different genes, need also be gained in order to improve our understanding of the cumulative effect of genetic variation on the risk of developing IA. In order for genetic screening of patients to be incorporated into routine clinical practice, automated pipelines will be required to perform genetic analysis, but also to calculate risk of developing IA based on complementary or confounding genetic and clinical risk factors and diagnostic results.

With many polymorphisms identified in single center studies, overcoming sample size, ethnical diversity and other clinical forms of population heterogeneity is critical, together with ensuring the influence of methodological, sample selection and statistical biases are minimized (van der Velden et al., 2011). Confidence in the significance of any association between a polymorphism and risk of IA is enhanced by the availability of multiple studies confirming the link.

Pentraxin-3 (PTX3) forms complexes on the surface of *Aspergillus* conidia where it acts as an opsonin by improved labelling of the microbe, resulting in enhanced phagocytosis and control of *Aspergillus* infection. Levels of PTX3 in the lung-alveolar fluid can be relatively low compared to other components capable of opsonizing *Aspergillus* conidia, but PTX3 genetic variants impair both alveolar expression of the protein and the antifungal effect mechanism of neutrophils (neutrophil extracellular traps), which play a primary role in the innate response during hyphal tissue invasion (Cunha and Carvalho, 2019). PTX3 has significant stimulus on pro-inflammatory cytokines and also interacts with many serum proteins involved the complement cascade but also CLRs, such as Dectin-1, with PTX3 potentially amplifying the Dectin-1 response (Cunha and Carvalho, 2019; Kang et al., 2020).

Polymorphisms in the PTX3 gene were first reported in 2014 where the homozygous haplotype (h2/h2) was associated with increased susceptibility to IA in allogeneic SCT patients in both the discovery and confirmatory study (**Table 1**) (Cunha et al., 2014). The haplotype resulted in defective PTX3 expression and ultimately resulted in impaired phagocytosis and fungal clearance. Since then several additional studies have confirmed the significance of this haplotype with increased risk of aspergillosis in SCT, solid organ transplantation recipients and patients with chronic respiratory conditions (Cunha et al., 2015; Wójtowicz et al., 2015; He et al., 2018). Its significance was also confirmed in the US study of Fisher and colleagues who screened for the presence of 20 SNP’s previously reported to be significantly associated with IA in 2609 SCT patients (483 with proven/probable IA) (Fisher et al., 2017). The number of independent studies corroborating the significance of this polymorphism across various ethnicities and clinical settings imply the presence pf PTX3 polymorphisms to be a robust indication of increased risk of aspergillosis.

A Spanish study screening 27 SNPs primarily in C-type lectin receptors (CLR) demonstrated six significant associations between polymorphisms in genes encoding Dectin-1 and DC-SIGN (**Table 1**) (Sainz et al., 2012). Dectin-1 recognizes (1-3)-β-D-Glucan, a major cell wall component of most fungi, while the other CLR (e.g. DC-SIGN, Dectin-2, Mannose Binding protein) primarily recognize mannose components of the fungal cell wall (Hardison and Brown, 2012). Ligand binding to these PRRs stimulates innate immune responses including fungal recognition, phagocytosis, induction of antifungal effector mechanisms and production of cytokines/chemokines, but also stimulates and controls the pro-inflammatory (T_H_1/T_H_17) adaptive immune response. The significance of two genetic variants in DC-SIGN (rs11465384 and rs7248637) and one in Dectin-1 (rs7309123) were confirmed in a UK study, with the two Dectin-1 SNPs (rs7309123 and rs3901533) also being confirmed in a recent Chinese study (White et al., 2017; Chen et al., 2019). DC-SIGN rs7248637 and Dectin-1 rs7309123 were also found to be associated with IA when defined cases of IA included proven/probable IA or patients that were positive by serum GM testing in the large US validation study (Fisher et al., 2017). A recent systematic review and meta-analysis confirmed the two Dectin-1 SNPs (rs7309123 and rs3901533) to be significantly associated with risk of IA (Zhou et al., 2019).

With their broad role in antimicrobial defense, genetic variations in TLRs have also been associated with increased susceptibility to IFD. While definitive, primary fungal ligands recognized by TLRs are still being identified, recognition of mannosylated cell wall components may play an important role in the innate immune response *via* the induction of pro-inflammatory cytokines (e.g. TNF-α and IL-6), neutrophil recruitment and fungal clearance (Salazar and Brown, 2018). It is unclear if TLRs play a primary role in human antifungal immunity, and they may have a collaborative role with CLRs or have increased responsibility in hosts with altered immunity. A significant link between the TLR4 haplotype (D299G/T399I) and increased risk of aspergillosis has also been confirmed in various studies including SCT and patients with chronic respiratory conditions (Bochud et al., 2008; Carvalho et al., 2008; de Boer et al., 2011; Koldehoff et al., 2013). While this provides confidence in the association between haplotype and aspergillosis, other studies contradict this finding, highlighting the difficulties in stratifying any approach screening for specific polymorphisms (Carvalho et al., 2009; Fisher et al., 2017).

Conflicting studies where the significance of polymorphisms deemed previously relevant is questioned, immediately focus attention on the few confirmed SNPS, but while unconfirmed SNPs could reflect limitations of the initial study, it could reflect ethnicity/evolutionary/clinical differences in the populations studied, and SNPs with conflicting results should not be immediately discarded without evaluation in large scale trials.

Of the two largest studies to date, one performed an extensive validation study of polymorphisms previously reported to be associated with IA in hematology patients. Two of 20 SNPs (PTX3 rs2305619 and Dectin 1 rs16910526) had a significant association with proven/probable IA confirmed (Fisher et al., 2017). Further polymorphisms in genes encoding C-type lectin receptors (Dectin 1, DC-SIGN), TLR (TLR6), chemokines (CXCL10), cytokines (IFN-γ), immune receptors (TNFR1), Ca_2_^+^ binding protein (S100B) and fibrinolytic proteins (plasminogen) were confirmed significant when patients with serum GM positivity were considered cases, in addition to more stringently classified proven/probable IA. The AspBIOmics consortium identified 36 SNPs within 14 immunomodulatory genes that were associated with immune response, had laboratory evidence of a biological function and/or had been previously reported as significantly associated with infectious diseases (Lupiañez et al., 2015). A total of 781 hematology patients (149 with proven/probable IA) were screened for the presence of these SNPs over two phases, the latter used to confirm results. Polymorphisms in IL4R (rs2107356) and IL8 (rs2227307) were significantly associated with increased risk of IA, whereas polymorphisms in IL12B (rs3212227) and IFN-γ (rs2069705) reduced the risk of IA, with the effects IL4R and IFN-γ polymorphisms emphasized in the allogeneic patient.

Given the range of polymorphisms associated with increased risk of IA it is unlikely that a single polymorphism will provide the required clinical utility. Testing multiple high-risk genes for the presence of indicative SNP’s seems inevitable but also astute, where specific patterns of genetic polymorphisms rather than a single genetic variation may meet the desired clinical requirements for predicting IA (de Boer et al., 2011; White et al., 2017). Advancing the field based on current knowledge would indicate that testing for well characterized SNPs in of PTX3, TLR4 and C-type lectin receptors would be a good starting place for strategic screening. However, while a recent prospective study in an Asian hematology population screened for relevant SNP’s in these genes, the only significant association with increased risk of IA was with C genotype in IL10 rs1800896, possibly indicating ethnical influence (Tanpaibule et al., 2020). If there is a requirement to screen for excessive numbers (>20) of SNPs in each patient then performing white cell functionality (human biomarker) studies to assess and individual’s peripheral blood mononuclear cells in response to *Aspergillus* stimuli might be an alternative approach (Zoran et al., 2022; Griffiths et al., 2021).

## Models Incorporating Genomics To Predict IA

Various predictive risk models of IA have been proposed, incorporating a wide array of clinical parameters (underlying condition/immune status/influence of treatment of underlying condition/co-morbidities/environmental and occupational risk) (Stanzani and Lewis, 2018). The D-index based on the duration and severity of neutropenia has shown to be sensitive (91-100%) by not necessarily specific (58%) for predicting IA (Portugal et al., 2009). Risk scores and indices for IFD have been proposed, but can be restricted to specific patient populations, lack prospective evaluation or have potential study design limitations (Stanzani and Lewis, 2018). A weighted risk score (BOSCORE) model for Hematology patients, initially incorporating four clinical risk factors (Active malignancy, prolonged neutropenia, severe lymphocytopenia and history of IFD) and recalibrated with additional risk factors (high dose steroids, high-risk chemotherapy, CMV reactivation/disease) confirmed the benefit of these strategies for excluding IFD (NPV>96%) and can be used to highlight patients requiring posaconazole prophylaxis, biomarker screening and or investigative bronchoscopy and chest CT (Stanzani and Lewis, 2018).

As with biomarker screening, predictive models based on clinical risk alone appear suitable for identifying patients at low risk of developing or without IA. While these models do highlight the patient with a higher risk of developing IA, significant numbers of high risk patients do not develop IA, limiting confidence in the strategy for confirming or pre-empting a diagnosis. With the number of clinical risk factors associated with risk of IFD increasing [e.g. immunomodulatory therapies, (etanercept)], models will require continual recalibration (Zoran et al., 2019).

**Figure 1:**
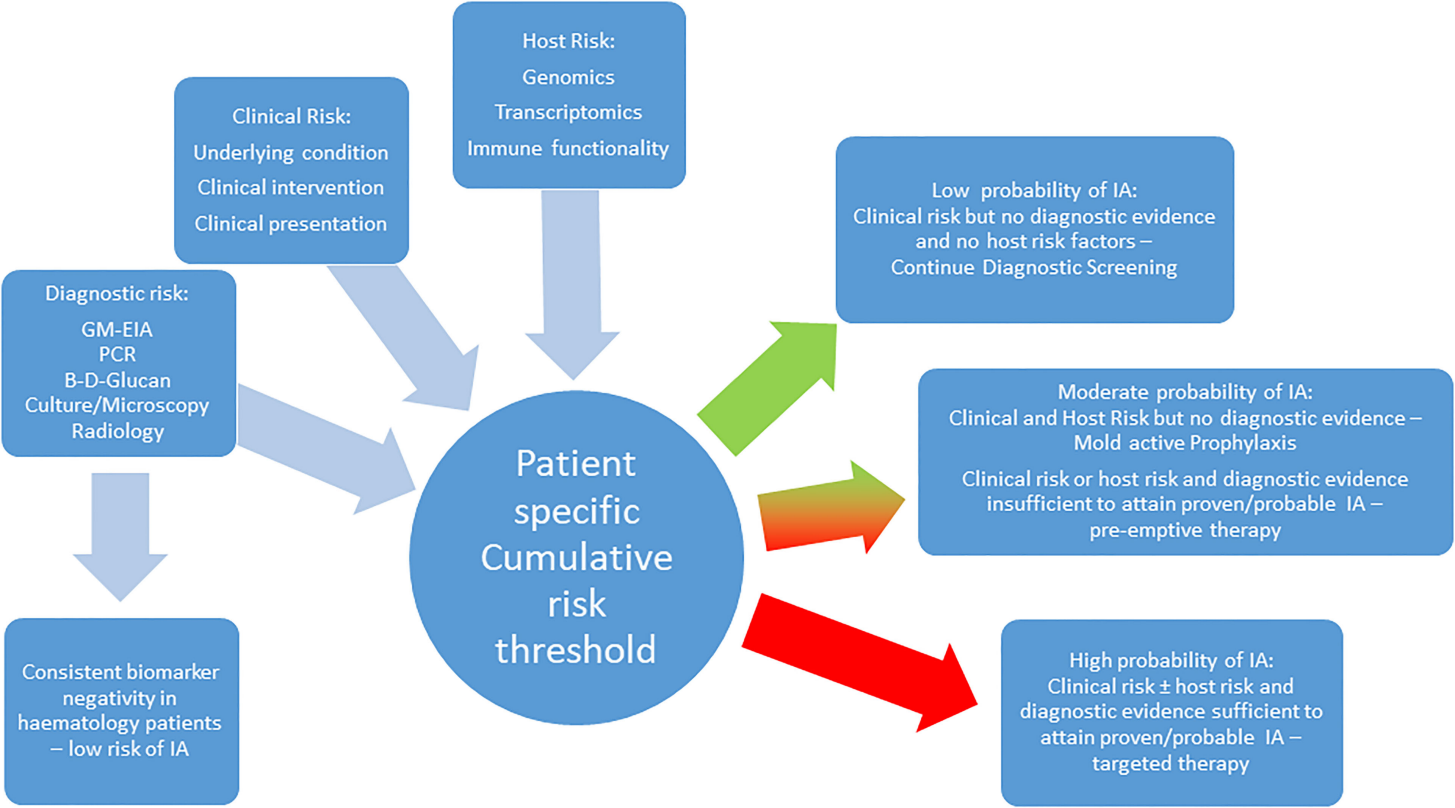
A proposed combined strategy incorporating clinical, host and diagnostic risk for managing the risk of IA in the hematology patient.

The association between polymorphisms in genes encoding proteins involved the host’s immune response and risk of aspergillosis appears undisputed, but judging the weight of risk associated with each polymorphism alone or when combined with other genetic variations or even clinical risk factors is essential but currently limited. While odds or hazard ratios are a statistically sound way of reporting significance, clinical interpretation is dependent on knowing the probability of IA developing in a patient with sufficient genomic and clinical risk, potentially combined with evidence of infection (**Figure 1**).

An understanding of how best to interpret the presence or, indeed, absence of a polymorphism is also important. The Dectin-1 variant rs7309123 has been associated with IA in several studies, yet it can be present in a significant proportion (65%) of hematology patients without IA and likely represents the major allele (White et al., 2017). So while the presence of this particular SNP is significantly associated with increased risk of IA, present in 87% of patients with IA in the referenced study (OR: 3.7 95% CI: 1.5-9.3), the subsequent diagnostic performance parameters [positive predictive value (PPV): 21%; negative predictive value (NPV): 93%] indicate the best way to use the polymorphism is to consider a reduced risk of, or resistance to IA in the absence of the major allele.

Conversely, other well characterized polymorphisms (DC-SIGN rs11465384 and rs7248637) can be much less evident in the patient without IA (11-14%) and while only present in 28-33% of patients with IA, the association between SNPs and IA remains significant (P: 0.001-0.004). Subsequent diagnostic performance parameters place the emphasis on increased risk of IA in the presence of the SNPs (specificity 86-89%), but not sufficiently to accurate predict IA (PPV: 33%) solely on the presence of these polymorphisms (White et al., 2017). While looking for the presence of a combination of SNPs (e.g. Dectin-1 rs7309123, DC-SIGN rs11465384 and rs7248637) previously independently associated with IA can increase the significance of the association (OR: 4.5, 95% CI: 2.2-9.2, P<0.001), the probability of predicting IA is only marginally improved (PPV: 39%). Given the presence of multiple positive biomarkers (*Aspergillus* PCR and GM) results already provide a probability of IA superior to this (PPV: 55%) shows that polymorphisms alone will not provide sufficient confidence for pre-empting or diagnosing IA (Barnes et al., 2013). Combining genetic screening with clinical risk and diagnostic testing is necessary, and may be further enhanced by monitoring the host’s RNA expression levels (transcriptomics) and immune functionality (proteomics/metabolomics) in response to stimulus by *Aspergillus* and its antigens (**Figure 1**).

Through stepwise logistic regression analysis, the AspBIOmics consortium developed a predictive model incorporating significant SNPs (IFN-γ, IL8, IL12p70 and VEGFA) with demographic and clinical risk factors for IA (age, gender, allogeneic SCT, anti-fungal prophylaxis) and compared the discriminatory capacity of this model with a reference model incorporating only demographic/clinical risk factors (Lupiañez et al., 2015). Through received operator characteristic (ROC) analysis the combined SNP/Demographic/Clinical model was shown to provide improved discrimination [Area under curve (AUC): 0.659] over the reference model (AUC: 0.564), highlighting the benefit of incorporating genomics. However, the improved AUC of the combined predictive model can still only be considered of moderate diagnostic capacity and using a threshold to achieve a specificity of 90% would only provide a sensitivity of <30% and *vice versa* (Lupiañez et al., 2015).

Taking this approach further, one retrospective study combined clinical and genetic risk with mycological evidence of IA in 322 hematology patients, 54 with proven/probable IA (White et al., 2017). Seven genetic markers previously associated with risk of IA were assessed and through stepwise multi-regression analysis a final model incorporating three SNPs (dectin-1 rs7309123, DC-SIGN rs11465384and rs7248637), two clinical risk factors (allogeneic SCT, prior respiratory virus infection) and one mycological test (*Aspergillus* PCR, GM being excluded as it was used to classify IA) was developed. In the absence of an allogeneic SCT, the risk of IA was low (<5% in 60% of patients without an allogenic SCT), with only 2% of this cohort being at >50% risk when all variables were met (White et al., 2017). The distribution was significantly different in the allogeneic SCT patient, with 96% of patients with risk >5% and 44% of patients with a risk >50%, when a patient was *Aspergillus* PCR positive with prior respiratory virus or multiple genetic risk markers. In 10% of allogeneic SCT patients who were *Aspergillus* PCR positive, with prior respiratory virus infection and multiple genetic risk markers the probability of IA was 90%. If the patient lacked any of the significant variables the probability of developing IA was <2%, whereas if four or more variables were present the probability of IA was 79%. An optimal threshold of three variables provided a mean probability of developing IA of 56.7%, compared to 6.3% in patients with <3 variables.

## Conclusions

IA in the high risk hematology patient can be confidently excluded though clinical risk modelling but more so combined biomarker screening. Through the combination of clinical and genetic risk with mycological evidence, we are approaching a time when we should be able to accurately predict the risk of IA in the hematology patient. Routine screening for genetic variation that increases risk of IA can help stratify patients, permitting targeted prophylaxis in high risk patients and help to determine the significance of fungal biomarker positivity in patients without overt clinical signs, improving the accuracy of administrating pre-emptive therapy. (**Figure 1**) It can identify genetic risk in patients that could be rectified by allogeneic SCT, where the donor’s genome lacks any significant polymorphisms associated with IA. Conversely, screening the donor’s genome can identify variants that will further increase the risk of IA post-SCT. However, inconsistencies in the association of specific SNPs with risk of IA need to be resolved through large scale, multi-center trials of promising candidate genes (e.g. PTX3, TLR-4, Dectin-1). Studies combining all parameters are limited and prospective evaluation of such models is required but is complicated by our expanding knowledge of genetic and clinical risks, which will drive the continual development of such modelling. Therefore it is essential that predictive models be automated and easily accessible to the clinician (e.g. BOSCORE - smartphone), which will allow developments in the field to be more easily incorporated but all also provide rapid and convenient results (Stanzani and Lewis, 2018). Understanding the host and their immune responses to infection through genomics, transcriptomics and metabolomics/proteomics is critical to achieving how we manage the individual’s risk of IA, underpinning personalized medicine.

## Author’s Note

This paper was submitted as part of the supplement on the Proceedings of the Mycology 2021 meeting.

## Author Contributions

Both authors made a significant contribution to the work reported, whether that is in the conception, study design, execution, acquisition of data, analysis and interpretation, or in all these areas; took part in drafting, revising or critically reviewing the article; gave final approval of the version to be published; have agreed on the journal to which the article has been submitted; and agree to be accountable for all aspects of the work.

## Conflict of Interest

PW: performed diagnostic evaluations and received meeting sponsorship from Bruker, Dynamiker, and Launch Diagnostics; received speaker fees, expert advice fees, and meeting sponsorship from Gilead; received speaker and expert advice fees from F2G and speaker fees MSD and Pfizer; and is a founding member of the European Aspergillus PCR Initiative.

The remaining author declares that the research was conducted in the absence of any commercial or financial relationships that could be construed as a potential conflict of interest.

## Publisher’s Note

All claims expressed in this article are solely those of the authors and do not necessarily represent those of their affiliated organizations, or those of the publisher, the editors and the reviewers. Any product that may be evaluated in this article, or claim that may be made by its manufacturer, is not guaranteed or endorsed by the publisher.
